# Navigating the intricate links between migration, climate change, and food insecurity in Latin America and the Caribbean

**DOI:** 10.1016/j.lana.2024.100967

**Published:** 2024-12-11

**Authors:** The Lancet Regional Health – Americas

Dec 18 marks the International Migrants Day proclaimed by the UN in 2000. The act of migrating has always been part of humanity; however, over the years, the complexities entailing this movement have increased, partly due to conflicts, political instability, inequalities, and climate change. As the world evolves, emerging trends in socioeconomic transformations that drive migration provide valuable insights into global changes and inform future planning strategies. In this December issue we present a special collection on Migration and Health in Latin America and the Caribbean (LAC) addressing issues related to stigma and discrimination; gender and intersectionality; climate change; and the response of health systems to the COVID-19 pandemic in Mexico, Colombia, and Peru. Migrants and people on the move face a myriad of other challenges as part of a complex conjuncture, one of the most pressing being food insecurity, defined as irregular access to safe and nutritious food for healthy growth and development, possibly leading to psychological and mental health outcomes. The number of people experiencing acute food insecurity rose from 135 million in 2019 to 345 million in 2022 in 82 countries, whereas in Latin America around 57 million people were affected by hunger. Thus, addressing the intertwined relationship between food insecurity and migration without delay is crucial.

The severity of the climate crisis is increasing each year, impacting people's lives and putting them at risk, leading to migration and displacement as consequences to the severe events. The LAC region is facing numerous challenges that affect migration dynamics. For example, in Brazil, floods resulted in more than 700 ,000 displaced individuals in 2022, and rain and floods were responsible for another 281, 000 in Colombia in the same year. Venezuela is one of the countries from the LAC region with the largest displaced population across borders, with more than 7 million refugees leaving their homes looking for safety and stability in other countries, such as Peru and Colombia. Extreme events associated with climate change can potentially affect food systems at every level of the supply chain. One of the main consequences of climate change is the exacerbation of food insecurity, leading to migration which in turn leads to more vulnerability to food insecurity.

Global food systems and greenhouse gas emissions are closely intertwined. Estimates indicate that the global food system is responsible for a third of greenhouse emissions, being the number one source of methane and loss of biodiversity. In terms of environmental degradation, the LAC region is facing many challenges and experiencing crises that affect crop fields in the region and disrupt people's livelihoods. The rise in food insecurity, fuelled by climate-related events such as heat waves, heavy rainfall, droughts, and wildfires, results in higher prices which in turn exacerbates food insecurity.

There are specific causes and effects related to human migration, with migrants encountering several difficulties during the active mobility period and also while settling into a new place. A cross-sectional study evaluating food insecurity in Venezuelan migrants and refugees residing in Peru established that four of ten households were facing food insecurity in 2022. Further, a scoping review showed that food insecurity was exacerbated in migrants during the COVID-19 pandemic due to economic hardships and insufficient government assistance in the LAC region, worsening their overall health and increasing inequalities.

Solutions related to agriculture and adaptation to new climate conditions are necessary and need to be addressed in the LAC region. When tackling food insecurity, one should keep in mind that improvement is not only about increasing food production but also should be about a responsive approach towards mobility, climate, and access. Measures—such as reducing carbon emissions, efficient and effective water usage, soil quality improvement, and increasing resilience to drought—should be a priority for governments; however, these are not enough to address food insecurity in the context of migration. Other approaches should take into account inclusive policies that incorporate lessons and knowledge from indigenous and local communities, focusing on the available resources to manage climate risks, and empowering communities and smallholder farmers with agricultural innovation and crisis management.

Migration is often characterised by conditions of poverty, discrimination, and unsatisfied basic needs. Trauma and stress follow migrants during the migration process, which can be accompanied by unhealthy conditions of transportation, hunger, and payment of high fees for displacement. This process can be further exacerbated when migrants arrive at their destination where they face difficulties in accessing food and basic goods that should be promptly made available by governments. In South America, the Southern Common Market (MERCOSUR) is an example of free movement arrangements made between countries in the region facilitating the immigration process and residence in participating countries. However, despite this arrangement, migrants face many difficulties when arriving in a new country, such as language barriers, absence of work, and economic instability. Public policies targeting migrants’ rights should be a priority, with resources allocated to improving the living and nutritional conditions, granting migration permits, enhancing working conditions, and ensuring access to appropriate health care.

The intricate links between migration, climate change, and food insecurity in LAC are complex and require a multifaceted approach. The region faces unique challenges, with prevalence of moderate or severe food insecurity which is higher than the global average. As the climate crisis intensifies, so will migration and food insecurity. Collaboration between governments, international organisations, and local communities are imperative to ensure the wellbeing and dignity of migrants, reduce their vulnerabilities, and ensure positive outcomes for origin and destination communities, thereby fulfilling human rights. 24 years has passed since the proclamation of International Migrants Day. It is now time to put people's health and quality of life as priorities.

